# Health-related quality of life of mothers of children with congenital heart disease in a sub-Saharan setting: cross-sectional comparative study

**DOI:** 10.1186/s13104-017-2856-6

**Published:** 2017-10-26

**Authors:** Lidia Sileshi, Endale Tefera

**Affiliations:** 1 Department of Pediatrics & Child Health, School of Medicine, Wolaita Sodo University, Wolaita Sodo, Ethiopia; 2 Department of Pediatrics & Child Health, Cardiology Division, School of Medicine, Addis Ababa University, P.O.Box 1768, Addis Ababa, Ethiopia

**Keywords:** Health-related quality of life, Congenital heart disease, Short Form-36, Mothers’ quality of life

## Abstract

**Background:**

While the Health Related Quality of Life of the children with congenital heart defects is primarily affected, caring for a child with birth defect has an impact on the family’s quality of life as well. Understanding the level of quality of life of the parents, which is likely to vary in different cultural settings, beliefs and parental educational status may help to implement educational programs and other interventional measures that may improve the HRQOL of parents of such children. This cross-sectional comparative study reports the health-related quality of life of mothers of children with congenital heart diseases in a sub-Saharan setting.

**Results:**

Mean age of the mothers in the study group was 32.2 ± 7.1 years where as that of the control group was 30.5 ± 6.5 years (*p* = .054). One hundred-four children had congenital cardiac lesions classified as mild to moderate while 31 patients had severe lesions. On average, mothers in the study group showed poor performance on the Short Form-36 (SF-36) with statistically significant differences on all sub-scales including general health perception, physical functioning, role physical, role emotional, social functioning, bodily pain, vitality and mental health. Severity of the congenital heart defect was not associated with statistically significant difference in the health-related quality of life of the mothers.

**Conclusions:**

Mothers of children with congenital heart disease in our study have significantly lower quality of life in all domains of SF-36 compared to the control group. Planning and devising a strategy to support these mothers may need to be part of management and clinical care of children with congenital heart diseases.

## Background

The most commonly reported incidence of congenital heart defects is between 4 and 10 per 1000, clustering around 8 per 1000 live births [1, 2]. While the Health Related Quality of Life (HRQOL) of the children, adolescents and adults with congenital heart defects is primarily affected [3], caring for a child with birth defect has an impact on the family’s quality of life as well. Financial burden due to reasons such as traveling for care and medical costs, difficulty of interaction with those outside the family, emotional turmoil as a result of a sense of isolation because they feel different from their peers which increases their depression or mental health problems are some of the most important factors [4–6].

Understanding the level of quality of life of the parents, which is likely to vary in different cultural settings, beliefs and parental educational status may help to implement educational programs and other interventional measures that may improve the HRQOL of parents of such children [5]. In most sub-Saharan communities, child-care and upbringing is almost the sole responsibility of mothers. Furthermore, mothers may sometimes be blamed or discriminated for having a child with congenital birth defects, which in some cases may result in dissolution of marriage. This study sought to determine the HRQOL of mothers of children with congenital heart disease in a sub-Saharan setting where the access to definitive treatment is rarely available. We speculated that the added bad news that invasive treatments for congenital heart diseases are not readily/regularly available in country could make the level of anxiety of the mothers even worse.

## Methods

This was a cross-sectional study comparing the HRQOL of mothers of children with congenital heart diseases who came for regular cardiac follow-up at the Addis Ababa University hospital (Tikur Anbessa hospital) and mothers who visited the outpatient pediatric clinics for other acute illnesses. A sample of 135 mothers in each group was calculated using Sample sizes for two Independent samples, Continuous outcome, n = 2(Zδ/E)^2^, where δ (standard deviation) taken from another study was 16.71 and the margin of error (E) was taken to be within 4 points. Mothers were included in the study after written consent was obtained. Consecutive mothers coming to the clinics From February 1 to April 30, 2015 were included in the sample.

The standard SF-36 was used to collect the data. The SF-36 consists of 36 items (questions) measuring physical and mental health status in relation to 8 health concepts: physical functioning, role limitations due to physical health, bodily pain, general health perceptions, vitality (energy/fatigue), social functioning, role limitations due to emotional health, general mental health (psychological distress/wellbeing). The questionnaire was translated into Amharic, a working language in the country with a view of using translators in case the mothers prefer any other local language. However, the participants included in this study by chance spoke the language avoiding the need for translators. The questionnaire was also adapted in a way it fits the cultural background of the society. The principal investigator trained the data collectors on how to use the form and how to collect the data. Data was collected in an interview with trained data collectors. Data on age, educational level, marital status, monthly income, general health, physical functioning and others was collected.

### Statistical methods

Data was entered into SPSS version 20 for windows and analyzed. Demographic variables were analyzed using descriptive statistics. Continuous variables were displayed as mean ± standard deviation. HRQOL parameters were compared between the two groups of mothers using t-test for two independent samples. Significance was taken to be at a p value of < .05.

## Results

Mean age of the mothers in the study group was 32.2 ± 7.1 years where as that of the control group was 30.5 ± 6.5 years (p value = .054). Socio-demographic data of the study and control groups is shown on Table 1. Regarding the distribution of congenital heart diseases in the children of mothers in the study group Ventricular Septal Defect (VSD) was the most common. Overall, 104 of the children belonging to mothers in the study group had mild to moderate lesions while 31 children had severe forms of congenital heart diseases (Table 2).

**Table 1:** Socio-demographic characteristics of study group and control groups

	Study group		Control group	
	Frequency	Percent	Frequency	Percent
Marital status				
Single	4	2.9	12	8.9
Married	119	88.1	112	82.9
Divorced	10	7.4	8	5.9
Widowed	2	1.5	3	2.2
Age (years)				
18–25 (22.9 ± 2.0 years)	26	19.3	31	22.9
26–35 (30.5 ± 3.1 years)	74	54.8	83	61.5
≥ 36 (41.2 ± 4.2 years)	35	25.9	21	15.6
Educational level				
Illiterate	19	14	28	20.7
Read and write	3	2.2	22	16.2
Primary school	44	32.6	48	35.5
≥ Secondary school education	69	51.1	37	27.4
Occupation				
House wife	91	67.4	72	53.3
Working	44	32.6	63	46.7

**Table 2:** Types of congenital heart diseases in the children of the study group

Mild to moderate lesions	Number	Severe lesions	Number
Ventricular septal defects (VSD) of different sizes	44	Tetralogy of Fallot	17
Patent ductus arteriosus (PDA)	19	Complete atrioventricular septal defect (AVSD)	5
Atrial septal defects (ASD)	13	Transposition of the great arteries (TGA)	4
Coarctation of the aorta	4	Double outlet right ventricle (DORV)	2
Pulmonary valve stenosis	3	Total anomalous pulmonary venous return (TAPVR)	2
Mitral valve prolapse/mitral regurgitation	4	Ebstein’s anomaly	1
Membranous subaortic stenosis	1		
Combinations of the above lesions	16		
Total	104	Total	31

On average, mothers in the study group showed poor performance on the SF-36 with statistically significant differences on all sub-scales including general health perception, physical functioning, role physical, role emotional etc. Table 3 shows comparison of the performance of mothers in the study group and the control group on SF-36 sub-scales.

**Table 3:** Performance of the study group on SF-36 sub-scale compared to the control group

	SF-36 sub-scale	Study group (Mean ± SD)	Control group (Mean ± SD)	p value
1	General health perception	66.0 ± 26.2	79.2 ± 10.9	< .001
2	Physical functioning	83.9 ± 22.8	90.7 ± 10.9	.002
3	Role physical	74.3 ± 53.7	85.4 ± 22.7	.028
4	Role emotional	55.5 ± 42.8	85.5 ± 24.3	< .001
5	Social functioning	65.0 ± 29.5	79.5 ± 15.9	< .001
6	Bodily pain	63.5 ± 26.5	77.0 ± 13.2	< .001
7	Vitality	60.6 ± 25.7	78.5 ± 11.2	< .001
8	Mental health	52.7 ± 19.1	78.6 ± 11.6	< .001

When the sub-group of mothers of children with severe congenital cardiac lesions was compared with those having children with mild to moderate congenital cardiac lesions (Table 4), there was no statistically significant difference in any of the sub-scales of SF-36.

**Table 4:** Performance of sub-group of mothers of children having severe CHD on SF-36 sub-scales compared with sub-group of mothers having children with mild to moderate CHD

SF-36 sub-scale		Mild to moderate CHD (n = 104) (Mean ± SD)	Severe CHD group (n = 31) (Mean ± SD)	p value
1	General health perception	66.4 ± 25.5	64.7 ± 29.0	.755
2	Physical functioning	83.9 ± 22.8	83.8 ± 23.4	.988
3	Role physical	75.2 ± 58.2	70.8 ± 33.5	.693
4	Role emotional	56.5 ± 42.4	51.9 ± 44.5	.605
5	Social functioning	70.0 ± 29.4	61.8 ± 30.0	.502
6	Bodily pain	63.5 ± 26.8	63.9 ± 25.9	.939
7	Vitality	60.1 ± 25.0	62.4 ± 28.3	.659
8	Mental health	53.2 ± 19.8	50.7 ± 16.9	.519

*CHD* congenital heart disease, *SD* standard deviation

## Discussion

Our study shows that mothers with children having congenital heart disease have a lower performance compared with mothers of children having minor acute illnesses in all domains of health-related quality of life. Other studies have also demonstrated that parents of children with heart diseases or other chronic illnesses may have higher level of stress compared with parents of children with other minor illnesses [5, 7–10]. They are generally at a high risk of anxiety, distress and hopelessness [9]. Studies have also shown that mothers have a more severe depression and emotional reactions compared to fathers [11]. This may even be more important in sub-Saharan settings where childcare and upbringing is mainly responsibility of the mothers and mothers have less social support and less access to information regarding their children’s illnesses. Social support and access to information has been shown to positively impact quality of life of such mothers. Besides, the unavailability of cardiac surgery or percutaneous interventions for congenital heart diseases in this part of the world is likely to play a role in adversely affecting the quality of life of mothers in various ways: (1) the news that the clinical condition is not treatable in country or that the treatment is very expensive may create a sense of frustration; (2) unoperated congenital heart diseases may lead to frequent clinical decompensation, requiring frequent clinical consultation and marked limitation of the child’s physical activity; (3) some untreated congenital heart diseases may be associated with failure to thrive and/or developmental delay, adding to the concern and worry of the mothers.

Addressing quality of life of mothers is important in that maternal depression and anxiety can adversely affect the care of a child with chronic condition like congenital heart diseases [12, 13]. Parents, especially mothers may need continuous psychosocial support before or after the child is treated as some studies indicate that the psychological stress and impaired quality of life may continue long after the child has undergone corrective surgery or invasive treatment [14].

Though some studies have suggested that maternal symptoms of depression and anxiety is related to severity of the congenital cardiac lesion [6], our study did not demonstrate any difference in any of the domains between those with mild to moderate and severe cardiac lesions. DeMaso et al. [13] have also shown that diagnosis of a severe cyanotic heart defect does not appear to make a child more likely to have emotional disorder compared to the ones with non-severe, in the absence of other factors. However, it is also possible that our finding may probably be related to the differences in the study settings, knowledge of mothers regarding the treatment and prognosis of each of the cardiac lesions, scarcity of intervention for any of the congenital cardiac lesions in our study setting and other related factors compared to those studies which concluded that severity of illness was important.

## Conclusions

Mothers of children with congenital heart disease in our study have significantly lower quality of life in all domains of SF-36 compared to the control group. Planning and devising a strategy to support these mothers may need to be part of management and clinical care of children with congenital heart diseases.

## Abbreviations

CHD: congenital heart disease
SF-36: Short Form-36
SPSS: statistical package for social sciences
VSD: ventricular septal defect
ASD: atrial septal defect
PDA: patent ductus arteriosus
AVSD: atrioventricular septal defect
TGA: transposition of the great arteries
DORV: double outlet right ventricle
TAPVR: total anomalous pulmonary venous return
SD: standard deviation
